# The Intersection of Non-Communicable Chronic Disease and Endodontic Care: A Pilot Retrospective Cross-Sectional Study

**DOI:** 10.3390/dj14020074

**Published:** 2026-02-02

**Authors:** Fausto Zamparini, Mohamed Mowafy, Andrea Spinelli, Stefano Chersoni, Igor Diemberger, Antonella Polimeni, Maria Giovanna Gandolfi, Carlo Prati

**Affiliations:** 1 Endodontic Clinical Section, Department of Biomedical and Neuromotor Sciences (DIBINEM), Dental School, University of Bologna, 40125 Bologna, Italy; fausto.zamparini2@unibo.it (F.Z.);; 2 Laboratory of Green Biomaterials and Oral Pathology, Department of Biomedical and Neuromotor Sciences, Dental School, University of Bologna, 40125 Bologna, Italy; 3 Department of Statistics and Quantitative Methods, University of Milano-Bicocca, Piazza dell’Ateneo Nuovo 1, 20126 Milan, Italy; 4 Cardiology Unit, IRCCS Azienda Ospedaliero-Universitaria di Bologna, 40125 Bologna, Italy; 5 Department of Oral and Maxillofacial Sciences, Sapienza University of Rome, Via Caserta 6, 00161 Rome, Italy

**Keywords:** endodontic therapy, systemic chronic diseases, comorbidity, cardiovascular disease, diabetes mellitus, polypharmacy, aging population, treatment planning, prognosis, retrospective study

## Abstract

**Objective:** To evaluate the prevalence of systemic chronic diseases among patients undergoing endodontic therapy in a University Department of Endodontology and to assess their potential implications for treatment planning and prognosis. **Methods:** A retrospective cross-sectional study analysis was performed on clinical records of patients aged > 18 years treated at the Department of Endodontics, University of Bologna. Data collected included demographic information, presence of chronic systemic diseases, endodontic disease and medication history. Descriptive statistics were used to estimate prevalence rates. **Results:** More than one third of patients (35%) presented with at least one systemic chronic disease or multiple comorbidities. Cardiovascular diseases (19.8%) were the most prevalent. Polypharmacy was observed in 32% of patients. Patients aged 40 years and older showed a significantly higher prevalence of systemic conditions compared to younger individuals. **Conclusions:** The study supports the finding that a high percentage of patients undergoing endodontic therapies present systemic chronic diseases, multiple comorbid conditions and polypharmacy. It is important to assess these factors and to customize endodontic treatment and decision-making. These realities are likely to become even more pronounced in the coming years, as global population aging continues apace.

## 1. Introduction

Chronic non-communicable diseases (NCDs) represent the leading cause of morbidity and mortality worldwide, accounting for more than 70% of global deaths [1]. The number of patients affected by NCDs has dramatically increased in recent years and involves a large proportion of the middle-aged and elderly population [2]. The global burden of NCDs, including cardiovascular disease, diabetes mellitus, respiratory disease, and metabolic disorders, is projected to rise further as life expectancy increases.

In patients affected by NCDs, decision-making and the type of endodontic treatment could be more difficult. The chronic assumption of multiple drugs (polypharmacy) may further complicate therapy, creating conditions that negatively influence healing capacity and increase the risk of treatment failure. Several systemic diseases, such as cardiovascular, endocrine/metabolic, and autoimmune disorders, have been associated with alterations in host immunity, tissue perfusion, and healing capacity. These factors may directly or indirectly contribute to the onset, persistence, or recurrence of endodontic lesions such as apical periodontitis. These conditions lead to significant oral modifications, leading to reduced salivary buffer ability, persistent soft tissue inflammation, impaired bone healing, persistent pain and bleeding [3,4,5,6]. Operative protocol must therefore be reconsidered and guided by systemic clinical parameters rather than dental considerations alone [1].

The prevalence of NCDs in a population requiring endodontic treatment is not well defined and is probably underestimated. It may be important to evaluate the percentage of patients affected by NCDs and their association with endodontic lesions.

Although the medical impact of NCDs has been extensively documented, their implications in dentistry—and particularly in endodontics—remain insufficiently defined and likely underestimated [3]. Endodontic therapy and tooth rehabilitation after root canal treatment often require multiple long appointments. Several chronic therapies could induce alteration of the endodontic affected tooth, with calcification, ankylosis, hypercementosis and pulp necrosis [7].

Endodontic therapy requires the use of anesthesia, rubber dam application and long time to disinfect, shape and fill the root canals. Further rehabilitation is required to stabilize and ensure biomechanical resistance to occlusal forces [6,8]. Data on the prevalence of systemic chronic conditions among patients specifically seeking endodontic treatment in academic clinical settings remain scarce. This lack of evidence limits our ability to design tailored protocols for risk management and long-term outcome prediction.

The aim of the present study was to evaluate the prevalence of systemic chronic diseases among patients attending a university endodontic clinic, and to assess their potential influence and impact on decision-making and type of therapy. Descriptive statistical analysis was performed to determine the prevalence of systemic chronic diseases and their distribution by age group.

## 2. Materials and Methods

### 2.1. Study Design

The study was designed as a retrospective cross-sectional study aiming to assess the prevalence of non-communicable chronic diseases and the corresponding use of pharmaceuticals. The investigation was approved by the local ethical committee (CE AVEC ENDO IMPLANT RETRO 10.22). This work was written according to the STROBE guidelines for observational epidemiological studies [9] and was conducted in full accordance with ethical principles, including the Declaration of Helsinki [10].

### 2.2. Data Collection and Management

Data from patients’ clinical records in one university dental clinic of the department of endodontics were extracted. All patients agreed through a signed informed consent at the moment of first visit. Patients aged more than 18 years, treated from 2024 to 2025 were included in the analysis only when presenting at least one permanent tooth with an endodontic pathology (pulpitis, pulp necrosis, re-exacerbated apical lesions). Vital pulp therapies, pulp capping or treatment of deciduous teeth were not included in the present investigation.

Each record includes a general medical history form completed by the patient during their first visit and periodically updated when necessary—such as after a long lapse since the last update or when a clinician identifies possible changes in the patient health status.

A random sample of records was drawn by one of the study authors, unaware of previous interventions. Relevant information—including demographics, chronic diseases, and medication use—was extracted and recorded into an Excel spreadsheet by the single clinician to ensure consistency in data collection.

The dataset was subsequently imported into the R statistical software version 4.3.1 (R Foundation for Statistical Computing, Vienna, Austria) for cleaning and preparation. Patient age was calculated based on their date of birth relative to the date of data extraction. All reported chronic conditions and medications were then grouped into clinically relevant categories to facilitate analysis.

Chronic conditions were then grouped into clinically meaningful categories based on organ system involvement and shared pathophysiological characteristics. Categories were not mutually exclusive, and patients could be assigned to more than one group if they presented with multiple chronic conditions.

Similarly, pharmacological treatments were classified into therapeutic categories according to their primary pharmacological indication and mechanism of action. Polypharmacy was defined when 2 or more chronic medications were recorded. As with chronic conditions, medication categories were not mutually exclusive, and patients could be assigned to more than one category if multiple treatments were reported.

### 2.3. Statistical Analysis

All statistical analyses were conducted using R software. Descriptive analyses were performed to summarize the demographic and clinical characteristics of the study population, including age, sex, smoking status, prevalence of chronic conditions, and medication use. These summaries were presented for the entire cohort and further stratified by clinical categories of diseases and medications.

To enhance interpretation, we employed a set of graphical and tabular visualizations. A tree map diagram was used to display the relative frequency of conditions within each diagnostic category, helping to identify the most common conditions and their distribution across categories. Grouping tables were constructed to show the distribution of the number of chronic conditions and medications consumed for each specific disease category across defined age groups.

Exploratory group comparisons of chronic conditions and medication use were performed across strata of age, sex, and smoking status to preliminarily explore the influence of these patient characteristics on the outcomes. Because the outcomes are count variables and did not meet the assumptions of normality, non-parametric tests were applied. Specifically, the Kruskal–Wallis test was used to compare outcomes across multiple age categories, while the Mann–Whitney U test (Wilcoxon rank-sum test) was applied for comparisons between two-group variables, including sex and smoking status.

To formally examine these relationships, we fit generalized linear models (GLMs) appropriate for count data. Poisson regression was used as the primary modeling approach, and in cases where overdispersion was detected, the Negative Binomial model was applied. Covariates included age, sex, and smoking status. To evaluate the robustness of the findings, we also conducted a sensitivity analysis using a restricted sample limited to individuals aged 30 to 79 years, excluding underrepresented groups at the extremes of the age distribution where outcome variability was limited.

To identify the critical age at which chronic disease burden and medication use increase most rapidly, we first aggregated the data by age and computed the mean count for each outcome. We then fitted generalized additive models (GAMs) with smooth functions of age to the age-aggregated means, using sample size at each age as weights. From these models, we extracted the first derivative of the age smoothly to quantify the rate of change. The age corresponding to the maximum positive first derivative was defined as the critical age, representing the point of greatest acceleration in chronic conditions and medication use. Finally, we generated dot plots with fitted regression curves to visually identify the critical age.

## 3. Results

### 3.1. Demographic Characteristics of the Analyzed Cohort

A total of 202 patients were analyzed. The sample consisted of 117 female patients (58%) and 85 males (42%). The mean age was 52.7 years old ± 18.4 indicating a notable spread of ages in our sample (Table 1). In fact, ages ranged from 18 to 87 years old, with the male population more dispersed than female population (18–87 vs. 20–84, respectively) and the female population marginally older than the male population (median: 56 vs. 54, respectively). A total of 38 participants were smokers or historical smokers (19%). Approximately two-thirds of the cohort (65%) did not report any chronic conditions. In contrast, 23% reported one chronic condition, 9.9% reported two, and 2.5% reported three or more chronic conditions. Regarding medication use, just over half of the participants reported no medication consumption (52%). Among the remainder, 17% reported using one medication, 8.9% reported two, and 22% reported three or more medications. (Table 1).

**Figure 1:**
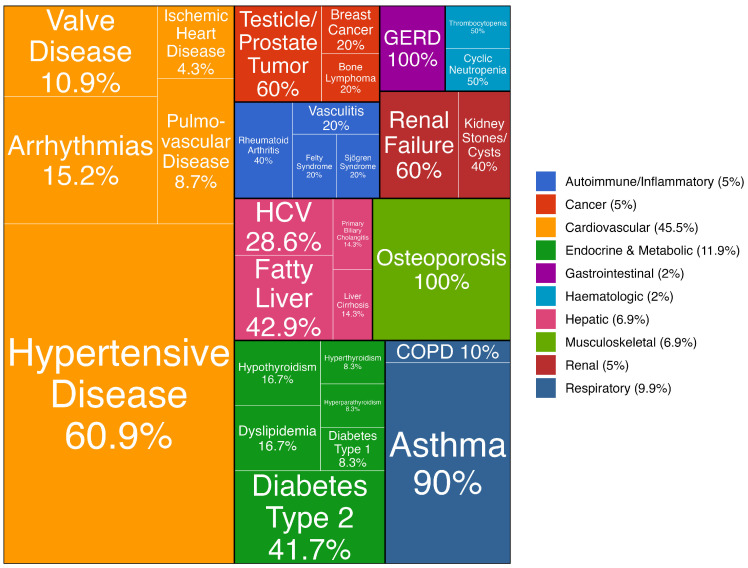
Proportional Treemap representation of chronic conditions among patients, grouped by disease category. Note: The musculoskeletal category consists exclusively of osteoporosis-related diagnoses in this sample.

Chronic conditions were categorized into eleven clinically meaningful groups: cardiocirculatory, endocrine/metabolic, autoimmune/inflammatory, oncologic, hematologic, hepatic, renal, respiratory, musculoskeletal, gastrointestinal, and neuro/psychiatric conditions. A tree map diagram (Figure 1) presents the relative distribution of each category and the proportion of individual conditions within each category. It is important to note that the percentages displayed in the diagram represent the frequency of each condition relative to others within the same category, rather than their overall prevalence in the cohort.

### 3.2. Chronic Conditions Within the Cohort

Table 2 presents the distribution of chronic conditions within the study cohort. Only the most prevalent conditions are shown individually; less common conditions, including gastrointestinal, hematologic, and neurologic conditions—have been grouped into a single category labeled “Other” chronic conditions. This composite group was reported by 4 patients (2.0%) and is further detailed in Supplementary Table S1. Notably, participants under the age of 20 did not report any chronic conditions.

Cardiocirculatory diseases were the most reported conditions, affecting 40 patients (19.8%). A higher proportion of females presented with these conditions compared to males (22.6% vs. 16.5%). Among females, the prevalence was highest in the 60–69 age group (55%), declining in the 70–79 age group (45.8%). In contrast, among males, the prevalence increased from 29.4% in those aged 60–69 to 64.6% in the 70–79 age group.

Respiratory conditions represented the second most prevalent category where at least one patient in every age group older than 20 and younger than 80 reported a respiratory condition, except those aged 40–49. 4.5% of the cohort suffered from Endocrine/metabolic conditions, specifically 5 (5.9%) male and 4 (3.4%) female patients. These conditions were most common in males aged 70–79 years (27.3%), with a lower prevalence observed among females of the same age group (8.3%).

Hepatic conditions were identified in 3.5% of the cohort, with the highest prevalence occurring among females aged 60–69 years (15%). Musculoskeletal disorders were represented exclusively by female patients and consisted solely of osteoporosis, which reached its peak prevalence in the 60–69 age group, affecting 20% of women in that category. Other chronic conditions were observed at comparatively lower frequencies, including renal disorders (2.5%), autoimmune diseases (2.5%), and oncologic conditions (2%).

### 3.3. Pharmaceutical Drug Consumption Within the Cohort

**Figure 2:**
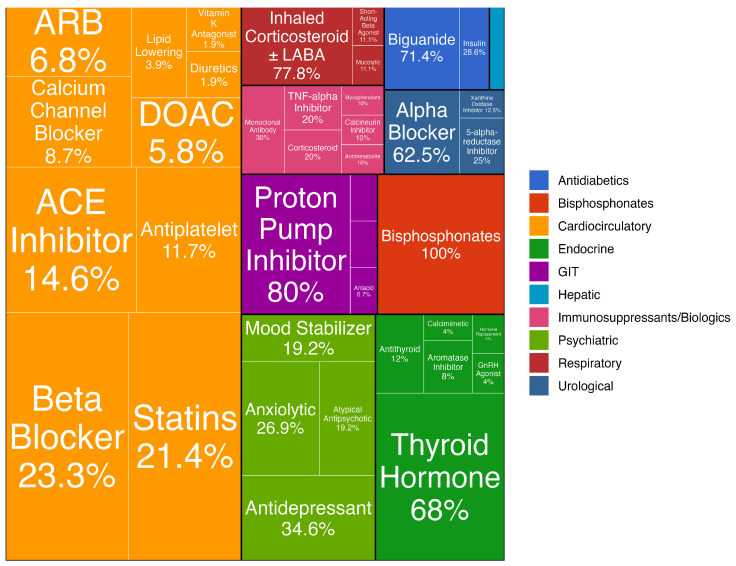
Proportional Treemap representation of pharmaceuticals consumed by patients, grouped by clinically meaningful categories. Note that 100% of hepatic drugs (cerulean box) were hepatic protective agents like ursobil.

Table 3 presents the distribution of pharmaceutical drug consumption across different therapeutic categories, stratified by sex and age groups. Medications targeting cardiocirculatory conditions represented the most frequently consumed drug class within the cohort, aligning with the most prevalent chronic conditions reported. A tree map diagram of pharmaceuticals consumed by patients is reported in Figure 2.

Notably, the proportion of patients consuming these medications (26.2%) exceeds the proportion diagnosed with corresponding cardiocirculatory conditions (19.8%), potentially reflecting their preventive or prophylactic use. Interestingly, male patients reported slightly higher consumption of these medications compared to females (27.1% vs. 25.6%), in contrast to the higher prevalence of cardiocirculatory conditions among females noted in Table 1. Nonetheless, the age-specific patterns of consumption were consistent with those observed in chronic condition prevalence: females aged 60–69 and males aged 70–79 demonstrated the highest rates of cardiocirculatory drug use (55% and 72.7%, respectively).

Psychiatric medications were used by 17.3% of the patients in our cohort, with a markedly higher proportion among females—almost twice that of their male counterparts (21.4% vs. 11.8%). The highest consumption rates among females were observed in those aged over 60 years, with the prevalence reaching its peak in the 70–79 age group (41.7%). Among males, the age group with the highest reported consumption was 50–59 years, where 25% of patients within that demographic reported use.

Medications prescribed for endocrine and metabolic conditions—primarily thyroid disorders and, to a lesser extent, diabetes types 1 and 2 and other hormonal therapies—were consumed by 12.4% of the cohort. Only four males over the age of 50 reported using medications in this category. In contrast, younger females—particularly those aged 20–29 years—demonstrated a comparatively high consumption rate (21.1%). Among older female age groups, use declined to approximately 15–20.8%, and in the 80+ group, only one of four female patients reported taking medications of this class.

Bisphosphonates were used by 6.4% of the study population. Among females, 10.3% reported bisphosphonate use, with a clear age-related increase: from 6.2% in the 40–49 age group to 25% in those aged 70–79. Only one male aged between 60 and 69 years, was found to use bisphosphonates.

Immunosuppressants and biologic agents had limited representation, with seven patients reporting consumption, exclusively in those aged 50 years or older. Gastrointestinal medications—predominantly proton pump inhibitors—were used by 6.4% of the cohort, with consumption highest among patients aged 60 years and older.

Additional drug categories, either of low relevance to the current clinical investigation or characterized by minimal usage, were grouped under a composite “Other” category, and are detailed in Supplementary Table S2.

### 3.4. Analysis of Chronic Conditions and Consumed Medication Across Demographic Variables

Group-level comparisons of the mean number of chronic conditions and medications consumed across age groups, sex, and smoking status are presented in Table 4. Statistically significant differences were observed in both the number of chronic conditions and the number of medications across age groups (*p* < 0.001). No significant differences were found between sex and smoking status groups for either chronic conditions or medication count (*p* > 0.1).

In order to understand the magnitude, direction and statistical significance of different patients’ characteristics on the count of chronic conditions and medications consumed by the cohort, we fitted multivariate regression models with the outcome being our count variables and the explanatory variables age, sex and smoking status. Since our outcome variable is a discrete count variable, we opted for a Poisson distribution generalized linear model (case with chronic count) and a negative binomial distribution in case of overdispersion (case with drug count). Despite observing a non-linear relationship between age and the count of chronic conditions, we opted for a Poisson generalized linear model (GLM) rather than a more complex generalized additive model (GAM) with a smoothed age fit.

Table 5 presents the results from multivariable count regression models evaluating the association between patient characteristics and the number of chronic conditions and medications consumed. Models were fitted on the full sample and on a restricted subset of participants aged 30 to 79 years. This sensitivity analysis was conducted to focus on the age groups with the greatest variability, as individuals younger than 30 or older than 79 were underrepresented and reported few chronic conditions or medications. In both the full and restricted models, male sex was associated with a lower rate of chronic conditions and medication use compared to females, although these differences were not statistically significant. Smoking status was associated with a higher rate of chronic conditions and a lower rate of medication use. However no statistical significance was reached.

Age demonstrated a consistent and significant association with both outcomes. In the full cohort, each additional year of age was associated with a 4.3% increase in the rate of chronic conditions and a 4% increase in the rate of medication use (*p* < 0.001). These associations were slightly stronger in the restricted sample, where each year of age corresponded to a 5.1% increase in the rate of chronic conditions and a 4.3% increase in the rate of medications consumed (*p* < 0.001 for both). The consistency of these findings across model specifications supports the robustness of age as a predictor in this context.

Despite the strength of age effects, the explanatory power of the models was modest, as indicated by McFadden’s R^2^ values ranging from 0.055 to 0.117. These results should therefore be interpreted as indicative of general trends rather than precise individual predictions.

### 3.5. Critical Age Determination

**Figure 3:**
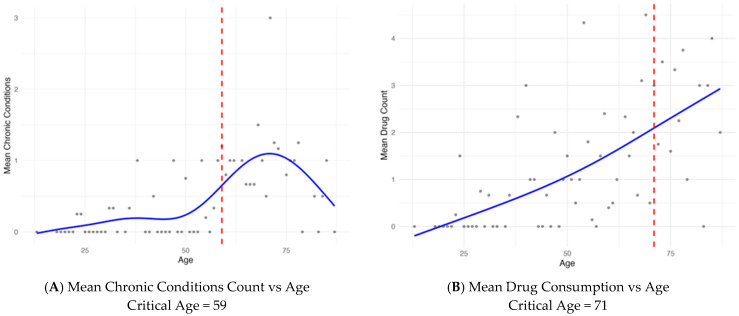
Association of age with (**A**) mean chronic conditions and (**B**) mean medication count, with fitted regression lines. Red line represents the critical age.

Figure 3 presents the fitted curves together with the corresponding critical ages, highlighted with a red dashed line: approximately 59 years for chronic conditions and 71 years for medication use. The data highlights the critical intersection between systemic non-communicable chronic diseases (NCDs) and endodontic therapy. A substantial proportion of patients—particularly those aged 35 years and above—presented with at least one systemic condition and undergoing chronic pharmacological treatment.

## 4. Discussion

The present study provided an overview of the systemic pathologies and pharmacological therapies present in a cohort of patients requiring endodontic treatment and supported the growing recognition that the systemic health status of patients must be considered a central factor in dental and endodontic decision-making.

The data reveals a substantial prevalence of chronic conditions and medications within this population. More than 30% of the cohort reported at least one pathology, and nearly half of the patients were taking one or more medications. Decision-making and intervention protocols may undergo significant variations based on medical pathologies [11]. Our data revealed that the most recurrent pathologies and related medications were cardiovascular diseases. Endodontic treatment should be modified in these patients (such as NYHA class III–IV). Previous investigations suggested that treatment should be rapid and minimally stressful, with effective pain control [12,13,14,15,16] and bleeding control.

Antibiotic prophylaxis should be prescribed for patients with a history of rheumatic heart disease, pacemakers, congenital heart disease, prosthetic cardiac valves, or vascular grafts due to the risk of transient bacteremia related to endodontic procedures, as rubber dam placement (9–32% of cases) and over instrumentation during root canal instrumentation (30–54%) [14,17]. Other less common conditions, observed in the present cohort, such as metabolic (4.5%) or musculoskeletal (osteoporosis-related) disorders (3.5%) had deep interconnections to endodontic treatment outcome, case complexities and post-operative complications. Patients suffering from diabetes mellitus usually report a higher prevalence of periapical lesions than health population. This population also show delayed wound healing and impaired immune responses with lower healing rates and higher occurrence of post-operative pain according to recent investigations [18,19,20,21].

Pharmacological management of systemic diseases further complicates clinical pictures [22]. Medications for cardiovascular diseases or hypertension (53 patients, 26.2% of the total cohort), such as beta-blockers, calcium channel blockers, ACE inhibitors, and diuretics (commonly prescribed for hypertension and reported in a high percentage of our cohort) are known to reduce salivary flow, diminishing the oral cavity natural protective mechanisms [23] and increasing the risk of carious lesions.

Other drugs, such as bisphosphonates or RANKL inhibitors (13 patients, 6.4% of the cohort) were linked to altered bone remodeling and osteonecrosis of the jaw [24,25,26]. These complications, although less frequent, require a deep modification of endodontic treatment protocols, focusing on minimal traumatic isolation techniques, careful use of local anesthesia, conservative instrumentation and obturation protocols to minimize apical trauma [27,28,29,30].

In the present study, demographic parameters showed clear associations with both chronic conditions and medication consumption. Descriptive analyses and multivariable regression models consistently demonstrated a significant, progressive increase in the number of chronic conditions and medications with advancing age. Each additional year of age was associated with an approximate 4–5% increase. The identification of critical ages—approximately 59 years for the accumulation of chronic conditions and 71 years for medication use—further illustrates the intersection between systemic health and endodontic therapy. Beyond these thresholds, a substantial proportion of patients present with multimorbidity and polypharmacy. Clinically, these critical ages may be used as stratification thresholds for preoperative assessment.

Smoking status (former smoker and actual smoker combined) showed a slight non-significant relationship with health. Smokers exhibited a higher rate of chronic conditions, but a lower rate of medication use compared with non-smokers, although these associations were not statistically significant. This is in accordance with recent investigations reporting a significant correlation between health-related quality of life and smoking status [31,32,33].

The study emphasized the concept that preoperative assessment should include evaluation of the patient’s systemic inflammatory burden, immunocompetence, and medication use. These realities are likely to become even more pronounced in the next years, as global population aging continues to increase worldwide.

Regarding the clinical protocols for endodontic treatments, it appears important to implement new single-file instrumentation protocols, efficient carrier based obturation protocols, likely associated with a biocompatible sealer. Single file techniques are nowadays promising as able to effectively remove necrotic tissues and endodontic smear layer from the root canals with low risk of fractures [34,35]. Carrier-based techniques are rapid, with a short learning curve but a valid long-term outcome (over 85% at 10-year follow-up) [36,37]. Premixed bioceramic sealers appear well tolerated by apical tissues confirmed by in vitro investigations [38,39] and according to systematic reviews [40,41].

Limitation of the study is related to the sample size, minimal adjustment for patient characteristics and modes power of McFadden’s R^2^ with values ranging from 0.055 to 0.117. The results should be interpreted as indicative of general trends rather than precise individual predictions. Additional limitation includes potential information bias due to the retrospective design of the study.

## 5. Conclusions

Given the increasing prevalence of patients affected by NCDs, endodontists must be adequately prepared to manage this growing population.

The identified critical ages (59 years for chronic conditions and 71 years for medication use) revealed groups in which multimorbidity and polypharmacy become more frequent and supports the importance of preoperative assessment in older adults requiring endodontic treatment.

Due to the relatively small sample size (n = 202), these findings should be considered preliminary and interpreted with caution, highlighting the need for further studies with larger cohorts.

## Figures and Tables

**Table 1:** Summary Statistics of Patients’ Demographics, Chronic Conditions Count and Pharmaceutical Consumption.

Characteristic	N = 202
Age	52.7 ± 18.4
Sex	
F	117 (58%)
M	85 (42%)
Smoker	38 (19%)
Chronic Disease Count	
0	131 (65%)
1	46 (23%)
2	20 (9.9%)
3+	5 (2.5%)
Drug Consumption	
0	105 (52%)
1	34 (17%)
2	18 (8.9%)
3+	45 (22%)

Notes: Continuous variables are reported as mean ± SD. Other variables are reported as count (%).

**Table 2:** Chronic Conditions Distribution Among Different Sex and Age Groups.

Chronic Condition	Total	<20	20–29	30–39	40–49	50–59	60–69	70–79	80+
**Cardiocirculatory**	**40 (19.8%)**								
Males	14 (16.5%)	-	-	-	-	2 (12.5%)	5 (29.4%)	7 (63.6%)	-
Females	26 (22.2%)	-	-	1 (12.5%)	-	1 (3.8%)	11 (55.0%)	11 (45.8%)	2 (50.0%)
**Musculoskeletal ^‡^**	**7 (3.5%)**								
Males	-	-	-	-	-	-	-	-	-
Females	7 (6.0%)	-	-	-	-	1 (3.8%)	3 (15.0%)	3 (12.5%)	-
**Endocrine/Metabolic**	**9 (4.5%)**								
Males	5 (5.9%)	-	-	-	-	1 (6.2%)	1 (5.9%)	3 (27.3%)	-
Females	4 (3.4%)	-	-	-	-	1 (3.8%)	1 (5.0%)	2 (8.3%)	-
**Respiratory**	**10 (5.0%)**								
Males	4 (4.7%)	-	-	2 (15.4%)	-	1 (6.2%)	1 (5.9%)	-	-
Females	6 (5.1%)	-	1 (5.3%)	-	-	2 (7.7%)	2 (10.0%)	1 (4.2%)	-
**Hepatic**	**7 (3.5%)**								
Males	3 (3.5%)	-	-	1 (7.7%)	-	-	2 (11.8%)	-	-
Females	4 (3.4%)	-	-	-	1 (6.2%)	-	3 (15.0%)	-	-
**Renal**	**5 (2.5%)**								
Males	3 (3.5%)	-	-	-	-	1 (6.2%)	-	2 (18.2%)	-
Females	2 (1.7%)	-	-	-	-	1 (3.8%)	1 (5.0%)	-	-
**Autoimmune**	**5 (2.5%)**								
Males	-	-	-	-	-	-	-	-	-
Females	5 (4.3%)	-	1 (5.3%)	1 (12.5%)	1 (6.2%)	-	1 (5.0%)	1 (4.2%)	-
**Oncologic**	**4 (2.0%)**								
Males	3 (3.5%)	-	-	-	-	-	1 (5.9%)	1 (9.1%)	1 (25.0%)
Females	1 (0.9%)	-	-	-	-	-	-	1 (4.2%)	-
**Other**	**4 (2.0%)**								
Males	2 (2.4%)	-	-	-	1 (9.1%)	-	1 (5.9%)	-	-
Females	2 (1.7%)	-	-	-	-	1 (3.8%)	1 (5.0%)	-	-

Note: Percentages in the Total row refer to the proportion of the entire study sample. Percentages in the Male and Female rows refer to the proportion within each sex. Percentages across age-group columns refer to the proportion within each age group for the corresponding row. ^‡^ All musculoskeletal diagnoses observed in the dataset correspond to osteoporosis-related conditions; no other musculoskeletal diseases were recorded.

**Table 3:** Distribution of Drug Consumption by Sex and Age Group in the Study Cohort.

Drug Category	Total	<20	20–29	30–39	40–49	50–59	60–69	70–79	80+
**Cardio/Circulatory**	**53 (26.2%)**								
Males	23 (27.1%)	-	-	-	1 (9.1%)	5 (31.2%)	7 (41.2%)	8 (72.7%)	2 (50.0%)
Females	30 (25.6%)	-	1 (5.3%)	1 (12.5%)	2 (12.5%)	2 (7.7%)	11 (55.0%)	11 (45.8%)	2 (50.0%)
**Bisphosphonates**	**13 (6.4%)**								
Males	1 (1.2%)	-	-	-	-	-	1 (5.9%)	-	-
Females	12 (10.3%)	-	-	-	1 (6.2%)	2 (7.7%)	3 (15.0%)	6 (25.0%)	-
**Antidiabetics**	**6 (3.0%)**								
Males	5 (5.9%)	-	-	-	-	1 (6.2%)	1 (5.9%)	3 (27.3%)	-
Females	1 (0.9%)	-	-	-	-	1 (3.8%)	-	-	-
**Endocrine**	**25 (12.4%)**								
Males	4 (4.7%)	-	-	-	-	1 (6.2%)	1 (5.9%)	1 (9.1%)	1 (25.0%)
Females	21 (17.9%)	-	4 (21.1%)	-	3 (18.8%)	5 (19.2%)	3 (15.0%)	5 (20.8%)	1 (25.0%)
**Gastrointestinal**	**13 (6.4%)**								
Males	6 (7.1%)	-	-	-	-	1 (6.2%)	2 (11.8%)	2 (18.2%)	1 (25.0%)
Females	7 (6.0%)	-	1 (5.3%)	-	1 (6.2%)	1 (3.8%)	1 (5.0%)	3 (12.5%)	-
**Psychiatric**	**35 (17.3%)**								
Males	10 (11.8%)	-	1 (10.0%)	1 (7.7%)	-	4 (25.0%)	2 (11.8%)	2 (18.2%)	-
Females	25 (21.4%)	-	1 (5.3%)	2 (25.0%)	2 (12.5%)	3 (11.5%)	6 (30.0%)	10 (41.7%)	1 (25.0%)
**Immunosuppressants/Biologics**	**7 (3.5%)**								
Males	2 (2.4%)	-	-	-	-	2 (12.5%)	-	-	-
Females	5 (4.3%)	-	-	-	-	-	3 (15.0%)	2 (8.3%)	-
**Other**	**32 (15.8%)**								
Males	12 (14.1%)	-	-	2 (15.4%)	-	3 (18.8%)	4 (23.5%)	1 (9.1%)	2 (50.0%)
Females	20 (17.1%)	-	2 (10.5%)	2 (25.0%)	3 (18.8%)	3 (11.5%)	2 (10.0%)	7 (29.2%)	1 (25.0%)

Note: Percentages in the Total row refer to the proportion of the entire study sample. Percentages in the Male and Female rows refer to the proportion within each sex. Percentages across age-group columns refer to the proportion within each age group for the corresponding row.

**Table 4:** Group comparisons of the mean number of chronic conditions and medications consumed, stratified by age category, sex, and smoking status.

Outcome Variable	Grouping Variable	Group 1	Group 2	Test	*p*-Value	Significance
**Chronic Count**	Age Category	NA	NA	K-W	2.7e-11	***
**Chronic Count**	Sex	M	F	Wilcox	0.75	.
**Chronic Count**	Smoker	No	Yes	Wilcox	0.66	.
**Drug Count**	Age Category	NA	NA	K-W	1.8e-07	***
**Drug Count**	Sex	M	F	Wilcox	0.19	.
**Drug Count**	Smoker	No	Yes	Wilcox	0.42	.

Signif. codes: ‘***’ < 0.001; ‘**’ < 0.05; ‘*’ < 0.1; ‘.’ > 0.1. Wilcox test used for binary variables (sex, smoking status); Kruskal–Wallis test used for categorical variables with more than two groups (age category).

**Table 5:** Comparison of regression coefficients, AIC, and McFadden’s R^2^ across four models estimating predictors of the number of chronic conditions and medications. For Poisson and Negative Binomial (NB) models, coefficients are presented as incidence rate ratios (IRRs), which can be interpreted as percentage increases or decreases in the expected count of outcomes associated with each predictor.

Variable	Chronic Count ^†^	Chronic Count ^†^ 30–79	Drug Count ^§^	Drug Count ^§^ 30–79
**(Intercept)**	0.044 ***	0.03 ***	0.148 ***	0.127 ***
**Age**	1.043 ***	1.051 ***	1.04 ***	1.043 ***
**Sex**	0.896	0.994	0.798	0.888
**Smoker**	1.284	1.157	0.726	0.535 .
**AIC**	365.78	326.82	588.87	507.75
**McFadden R^2^**	0.117	0.101	0.074	0.055
**Observations**	202	162	202	162

Signif. codes: ‘***’ <0.001; ‘**’ < 0.05; ‘*’ < 0.1; ‘.’ > 0.1. ^†^ Poisson regression (GLM) models; ^§^ Negative binomial regression (GLM.nb) models. Note: An IRR greater than 1 indicates an increase in the expected number of conditions or medications, while an IRR less than 1 indicates a decrease.

## Data Availability

The data presented in this study are available on request from the corresponding author. The data are not publicly available due to privacy or ethical restrictions.

## Supplementary Materials

The following supporting information can be downloaded at: https://www.mdpi.com/article/10.3390/dj14020074/s1, Table S1: Chronic Conditions Distribution Among Different Sex and Age Groups; Table S2: Distribution of Drug Consumption by Sex and Age Group in the Study Cohort.
