# Supplementary material for: The Intersection of Non-Communicable Chronic Disease and Endodontic Care: A Pilot Retrospective Cross-Sectional Study
Source: Dent J (Basel). 2026 Feb 2;14(2):74. doi: 10.3390/dj14020074 (PMC12939834; doi:10.3390/dj14020074)
Supplement: Supplementary file 1 [file dentistry-14-00074-s001.zip › dentistry-4096843-supplementary.pdf]

Supplementary Table 1: Chronic Conditions Distribution Among Different Sex and Age Groups

Chronic Condition	Total	<20	20-29	30-39	40-49	50-59	60-69	70-79	80+
**Cardiovascular**	**40 (19.8%)**								
Males	14 (16.5%)	-	-	-	-	2 (12.5%)	5 (29.4%)	7 (63.6%)	-
Females	26 (22.2%)	-	-	1 (12.5%)	-	1 (3.8%)	11 (55.0%)	11 (45.8%)	2 (50.0%)
**Musculoskeletal**	**8 (4.0%)**								
Males	-	-	-	-	-	-	-	-	-
Females	8 (6.8%)	-	-	-	-	1 (3.8%)	4 (20.0%)	3 (12.5%)	-
**Endocrine and Metabolic**	**9 (4.5%)**								
Males	5 (5.9%)	-	-	-	-	1 (6.2%)	1 (5.9%)	3 (27.3%)	-
Females	4 (3.4%)	-	-	-	-	1 (3.8%)	1 (5.0%)	2 (8.3%)	-
**Respiratory**	**9 (4.5%)**								
Males	3 (3.5%)	-	-	2 (15.4%)	-	1 (6.2%)	-	-	-
Females	6 (5.1%)	-	1 (5.3%)	-	-	2 (7.7%)	1 (5.0%)	2 (8.3%)	-
**Hepatic**	**7 (3.5%)**								
Males	3 (3.5%)	-	-	1 (7.7%)	-	-	2 (11.8%)	-	-
Females	4 (3.4%)	-	-	-	1 (6.2%)	-	3 (15.0%)	-	-
**Renal**	**5 (2.5%)**								
Males	3 (3.5%)	-	-	-	-	1 (6.2%)	-	2 (18.2%)	-
Females	2 (1.7%)	-	-	-	-	1 (3.8%)	1 (5.0%)	-	-
**Autoimmune**	**5 (2.5%)**								
Males	-	-	-	-	-	-	-	-	-
Females	5 (4.3%)	-	1 (5.3%)	1 (12.5%)	1 (6.2%)	-	1 (5.0%)	1 (4.2%)	-
**Gastrointestinal**	**2 (1.0%)**								
Males	2 (2.4%)	-	-	-	1 (9.1%)	-	1 (5.9%)	-	-
Females	-	-	-	-	-	-	-	-	-
**Haematological**	**2 (1.0%)**								
Males	1 (1.2%)	-	-	-	1 (9.1%)	-	-	-	-
Females	1 (0.9%)	-	-	-	-	1 (3.8%)	-	-	-
**Neurological**	**1 (0.5%)**								
Males	-	-	-	-	-	-	-	-	-
Females	1 (0.9%)	-	-	-	-	-	1 (5.0%)	-	-
**Oncological**	**4 (2.0%)**								
Males	3 (3.5%)	-	-	-	-	-	1 (5.9%)	1 (9.1%)	1 (25.0%)
Females	1 (0.9%)	-	-	-	-	-	-	1 (4.2%)	-

Supplementary Table 2: Distribution of Drug Consumption by Sex and Age Group in the Study Cohort

Drug Category	Total	<20	20-29	30-39	40-49	50-59	60-69	70-79	80+
**Cardio/Circulatory**	**53 (26.2%)**								
Males	23 (27.1%)	-	-	-	1 (9.1%)	5 (31.2%)	7 (41.2%)	8 (72.7%)	2 (50.0%)
Females	30 (25.6%)	-	1 (5.3%)	1 (12.5%)	2 (12.5%)	2 (7.7%)	11 (55.0%)	11 (45.8%)	2 (50.0%)
**Bisphosphonates**	**13 (6.4%)**								
Males	1 (1.2%)	-	-	-	-	-	1 (5.9%)	-	-
Females	12 (10.3%)	-	-	-	1 (6.2%)	2 (7.7%)	3 (15.0%)	6 (25.0%)	-
**Antidiabetics**	**6 (3.0%)**								
Males	5 (5.9%)	-	-	-	-	1 (6.2%)	1 (5.9%)	3 (27.3%)	-
Females	1 (0.9%)	-	-	-	-	1 (3.8%)	-	-	-
**Endocrine**	**25 (12.4%)**								
Males	4 (4.7%)	-	-	-	-	1 (6.2%)	1 (5.9%)	1 (9.1%)	1 (25.0%)
Females	21 (17.9%)	-	4 (21.1%)	-	3 (18.8%)	5 (19.2%)	3 (15.0%)	5 (20.8%)	1 (25.0%)
**Gastrointestinal**	**13 (6.4%)**								
Males	6 (7.1%)	-	-	-	-	1 (6.2%)	2 (11.8%)	2 (18.2%)	1 (25.0%)
Females	7 (6.0%)	-	1 (5.3%)	-	1 (6.2%)	1 (3.8%)	1 (5.0%)	3 (12.5%)	-
**Psychiatric**	**35 (17.3%)**								
Males	10 (11.8%)	-	1 (10.0%)	1 (7.7%)	-	4 (25.0%)	2 (11.8%)	2 (18.2%)	-
Females	25 (21.4%)	-	1 (5.3%)	2 (25.0%)	2 (12.5%)	3 (11.5%)	6 (30.0%)	10 (41.7%)	1 (25.0%)
**Immunosuppressants / Biologics**	**7 (3.5%)**								
Males	2 (2.4%)	-	-	-	-	2 (12.5%)	-	-	-
Females	5 (4.3%)	-	-	-	-	-	3 (15.0%)	2 (8.3%)	-
**Respiratory**	**7 (3.5%)**								
Males	2 (2.4%)	-	-	1 (7.7%)	-	1 (6.2%)	-	-	-
Females	5 (4.3%)	-	1 (5.3%)	1 (12.5%)	-	1 (3.8%)	-	1 (4.2%)	1 (25.0%)
**Hepatic**	**1 (0.5%)**								
Males	-	-	-	-	-	-	-	-	-
Females	1 (0.9%)	-	-	-	1 (6.2%)	-	-	-	-
**Urological**	**6 (3.0%)**								
Males	6 (7.1%)	-	-	-	-	1 (6.2%)	2 (11.8%)	1 (9.1%)	2 (50.0%)
Females	-	-	-	-	-	-	-	-	-
**Vitamins**	**14 (6.9%)**								
Males	4 (4.7%)	-	-	1 (7.7%)	-	2 (12.5%)	1 (5.9%)	-	-
Females	10 (8.5%)	-	-	1 (12.5%)	1 (6.2%)	2 (7.7%)	1 (5.0%)	5 (20.8%)	-
**Other**	**6 (3.0%)**								
Males	2 (2.4%)	-	-	-	-	-	2 (11.8%)	-	-
Females	4 (3.4%)	-	1 (5.3%)	-	1 (6.2%)	-	1 (5.0%)	1 (4.2%)	-
